# Characterization of the Psychrotrophic Lactic Acid Bacterium *Leuconostoc gelidum* subsp. *aenigmaticum* LS4 Isolated from Kimchi Based on Comparative Analyses of Its Genomic and Phenotypic Properties

**DOI:** 10.3390/foods10081899

**Published:** 2021-08-16

**Authors:** So Yeong Mun, Ye Jin Seo, Hae Choon Chang

**Affiliations:** Kimchi Research Center, Department of Food and Nutrition, Chosun University, 309 Pilmun-daero, Dong-gu, Gwangju 61452, Korea; ohno1757@gmail.com (S.Y.M.); yg0494@naver.com (Y.J.S.)

**Keywords:** kimchi, psychrotrophic, *Leuconostoc gelidum* subsp. *aenigmaticum*, starter culture

## Abstract

With the aim of developing a new food starter culture, twenty-three psychrotrophic lactic acid bacteria (LAB) were isolated from 16 kimchi samples. One strain, *Leuconostoc gelidum* subsp. *aenigmaticum* LS4, which had typical psychrotrophic characteristics, was selected, and its phenotypic and genomic properties as a starter culture were investigated. The complete genome of *L. aenigmaticum* LS4 consisted of one circular chromosome (1,988,425 bp) and two plasmids (19,308 bp and 11,283 bp), with a guanine–cytosine content of 36.8%. *L. aenigmaticum* LS4 could grow at 5 °C but not at 37 °C, and maximum cell growth was obtained at 15~25 °C. *L. aenigmaticum* LS4 did not show any harmful characteristics such as hemolysis, undesirable enzyme activities, biogenic amine production, or antibiotic resistance. *L. aenigmaticum* LS4 was investigated for its suitability for technological processes (pH, temperature and NaCl treatment). *L. aenigmaticum* LS4 exhibited strong antimicrobial activity caused by the production of organic acids and bacteriocin, and it produced an exopolysaccharide composed of glucose with a molecular weight of 3.7 × 10^6^ Da. Furthermore, *L. aenigmaticum* LS4 improved the organoleptic qualities of kimchi juice. Our results indicate that *L. aenigmaticum* LS4 could be used as a functional starter culture for food (vegetable or fruit) fermentation at low temperatures.

## 1. Introduction

Kimchi, a traditional Korean fermented vegetable, is well known for its health benefits, which include anticancer, anti-obesity, immune promotion, brain health promotion, cholesterol reduction, and antioxidative effects [1,2]. Numerous lactic acid bacteria (LAB) are involved in kimchi fermentation; thus, kimchi is considered a good source of potentially beneficial LAB [3]. The growing consumer demand for healthier foods is stimulating new product development in the food industry worldwide. Various LAB strains were isolated from kimchi with the aim of developing new probiotics or starter cultures for foods [3], but most of these isolates were mesophilic LAB due to the use of a 30 °C incubation temperature in isolation experiments [4,5,6]. However, kimchi is generally fermented at temperatures of less than 10 °C [7]; thus, it can be inferred that LAB that can grow well at low temperatures play an important role in kimchi fermentation.

Active LAB populations responsible for kimchi fermentation are of the genera *Lactobacillus*, *Leuconostoc*, or *Weissella* as determined by culture-independent and culture-dependent methods [8]. Thus far, LAB strains that grow well at 30 °C have been isolated, such as *Leuconostoc mesenteroides*, *Leuconostoc citreum*, *Leuconostoc kimchii*, *Lactobacillus plantarum*, *Lactobacillus sakei*, *Weissella confusa*, *Weissella cibaria*, and *Weissella koreensis* [3,9,10,11]. On the other hand, psychrotrophic LAB, which barely grow at 30 °C, have also been frequently identified in kimchi using culture-independent methods [8,12,13]; however, these LAB can rarely be isolated using routine mesophilic enumeration methods. Psychrotrophic LAB might play an important role in kimchi fermentation at low temperatures. However, little information is available about the phenotypic or genomic characteristics of psychrotrophic LAB isolated from kimchi. Specifically, at present, ten complete genome sequences (CGS) of psychrotrophic *L. gelidum* strains have been sequenced and deposited in GenBank: one CGS for *L. gelidum*, two CGSs for *L. gelidum* subsp. *gelidum*, and seven CGSs for *L. gelidum* subsp. *gasicomitatum* (https://www.ncbi.nlm.nih.gov/ (accessed on 24 May 2021)). Furthermore, no investigation has been conducted on the phenotypic characterization of *L. gelidum* subsp. *aenigmaticum*, although phenotypic characteristics were reported regarding the classification of *L. gelidum* subsp. *aenigmaticum* using a type strain of this subspecies [14]. Genetic information only provides potential phenotypic characteristics. Accordingly, more comprehensive studies about the phenotypic and genomic characteristics of new psychrotrophic LAB intended for use as starter cultures should be carried out.

In the present study, we isolated psychrotrophic LAB from kimchi using two different temperatures (25 °C or 30 °C), because psychrotrophs can grow optimally at 20~25 °C [15]. The isolated LAB were identified and then one LAB strain, *L. gelidum* subsp. *aenigmaticum*, was selected and investigated for its phenotypic characteristics, including safety, technological, and functional aspects, with a view toward using it in food starter cultures. Furthermore, we completed its genome sequence, analyzed its genomic properties, and then compared them with its phenotypic characteristics. We believe that the results of this study broaden the knowledge of the characteristics of psychrotrophic LAB in kimchi, about which relatively little is known as compared with mesophilic LAB.

## 2. Materials and Methods

### 2.1. Isolation and Identification of Psychrotrophic LAB from Kimchi

Sixteen kimchi samples stored at 0~10 °C for 1~4 months were collected from different locations in South Korea. Kimchi samples were macerated (hand blender, HHM-600; Hanil, Seoul, Korea) and filtered through a thin cloth, after which kimchi filtrates were diluted and spread onto de Man, Rogosa, and Sharpe (MRS; Difco, Sparks, MD, USA) agar supplemented with 2.0% CaCO_3_ [7]. Plates were incubated at 25 °C for 48 h; thereafter, each colony that formed a clear zone on the MRS + 2.0% CaCO_3_ agar was simultaneously toothpicked onto two MRS agars and incubated for 48 h at 25 °C or 30 °C. After incubation, colonies that grew at 25 °C but not at 30 °C were tentatively selected as psychrotrophic LAB and subjected to further experiments. The isolates were identified by morphological observations (Eclipse 55i, Nikon, Tokyo, Japan), Gram-staining, the catalase test, and by determining and comparing 16S rRNA sequences [16].

### 2.2. Polymerase Chain Reaction (PCR)-Denaturing Gradient Gel Electrophoresis (DGGE)

Microbial analysis of kimchi samples was also carried out using a culture-independent method, PCR-DGGE [7]. Briefly, DNA as a template for PCR was extracted using DNeasy Blood & Tissue Kits (Qiagen, Hilden, Germany), amplified using 27F and 1492R 16S universal primer, and then the V3 region of the 16S rRNA gene was amplified using *Lac1* and GC *Lac2* primers [2]. For DGGE analysis, we used 8% (*w/v*) polyacrylamide gel (BioRad, Hercules, CA, USA) at 50 V for 20 h. Bands of interest were extracted and reamplified using *Lac1* and GC *Lac2* primers. Thereafter, sequences of the amplified genes were determined using a DNA sequencer (Applied Biosystems, Forster City, CA, USA) and queried by BLAST searches of the GenBank database to identify closest known relatives.

### 2.3. Physiological and Biochemical Properties of LAB Isolates

#### 2.3.1. Growth at a NaCl Concentration of 6.5%

Growth of isolates in medium containing 6.5% (*w/v*) NaCl solid powder was determined, as previously described [14]. Briefly, isolates were inoculated (1.0%, *v/v*) into MRS broth containing 6.5% NaCl solid powder and then anaerobically incubated (HB-103S, Hanbaek, Bucheon, Korea) for 21 days at 25 °C. Growth was measured at 600 nm (A_600_; Ultrospec 2100 Pro, Biochrom, Cambridge, UK).

#### 2.3.2. Heme-Stimulated Aerobic Growth

Heme-stimulated aerobic growth of isolates was determined, as previously described [17]. Briefly, a stock solution of heme (0.5 mg/mL in 0.05 M NaOH; Sigma, St. Louis, MO, USA) was prepared and added to MRS broth to a final concentration of 2 µg/mL. LAB isolates were inoculated (1.0 %; *v/v*) into 50 mL of the heme-containing MRS broth in 250 mL flasks and incubated at 25 °C under aerobic conditions with shaking at 250 rpm (HK-S125C; Hankook, Hwaseong, Korea) for 48 h. Cultures without heme were used as controls.

#### 2.3.3. Carbohydrate Utilization

Carbohydrate utilization by the LAB isolates was determined using API 50 CHL (BioMérieux, Marcy l’Etoile, France), according to the manufacturer’s instructions. Isolates were inoculated into the API strip and incubated for 48 h at 25 °C, and acid production from the supplied carbohydrate was determined as described by the manufacturer.

#### 2.3.4. Growth at Low Temperature

LAB isolate growth was examined in MRS broth at 0, 5, 10, 15, 25, 30, or 37 °C for 0~240 h. During cultivation, growth was determined every 4, 8, or 24 h by measuring absorbance at 600 nm (A_600_; Biochrom).

### 2.4. Phenotypic Characteristics as Starter Cultures

#### 2.4.1. Sensory Properties

We evaluated the effects of the LAB isolates on the organoleptic qualities of kimchi juice. To prepare starter cultures, LAB isolates cultivated overnight in MRS were centrifuged at 9950× *g* for 15 min at 4 °C, washed with sterile distilled water, and resuspended in the same volumes of distilled water. To prepare kimchi juice, freshly prepared non-fermented kimchi was macerated using a juice maker (HD-RBF09, Hurom, Seoul, Korea), stored at −20 °C, and thawed under running tap water when required. The prepared starter cultures were inoculated at 5.0% (*v/v*) into prepared kimchi juice and fermented for 24 h at 25 °C; thereafter, its sensory characteristics were investigated. The kimchi juice without starter inoculum was used as a control.

Sensory evaluations were carried out after obtaining approval from the Institutional Review Board of Chosun University (IRB # 2-1041055-AB-N-01-2020-29). Nine trained graduate students, who had performed more than 30 sensory evaluations on kimchi per year at the Kimchi Research Center, Chosun University (Gwangju, Korea), conducted the evaluation. Samples (10 mL) labeled with a random 3-digit code were served on white bowls. The panelists evaluated sensory attributes using 5-point scales for sourness, fresh taste, carbonate taste, pleasant fermentative smell, sewerage-like smell (1 = very weak, 3 = moderate, and 5 = very strong), and overall acceptance (1 = very bad, 3 = moderate, and 5 = very good).

#### 2.4.2. Hemolysis and Antibiotic Resistance

Hemolysis was examined by streaking LAB cells on blood agar containing 7.0% horse blood (Oxoid, Hampshire, UK). Plates were incubated for 48 h at 25 °C to detect α-hemolysis or for 48 h at 25 °C and then kept at 4 °C for 24 h to detect β-hemolysis. The clear zones around colonies were then observed [9,18].

Antibiotic susceptibilities of LAB isolates were evaluated according to the technical guidelines issued by the European Food Safety Authority (EFSA) [19]. Minimal inhibitory concentrations (MICs) of ampicillin, chloramphenicol, erythromycin, gentamycin, kanamycin, streptomycin, tetracycline, and vancomycin (Sigma) were determined [9]. Briefly, LAB isolates cultivated overnight were centrifuged (9950× *g*, 15 min, 4 °C) and resuspended in Mueller–Hilton (MH; Difco) broth supplemented with 0.5% dextrose (final cell concentration; 7.0 log CFU/mL). Antibiotics were then added to aliquots of MH suspension and incubated anaerobically for 24~48 h at 25 °C [9]. Growth of LAB isolates was determined at 600 nm (Biochrom). MH cultures not treated with antibiotics were used as controls.

#### 2.4.3. Enzymatic Activities

API-ZYM kits (BioMérieux) were used to determine the enzymatic activities of LAB isolates. LAB were cultivated, harvested, and resuspended (McFarland standard 5) in sterile distilled water, and suspensions were then spotted (65 μL) into wells and incubated for 4 h at 25 °C. Then, one drop of the kit reagents ZYM-A and ZYM-B was then added to each well, and after allowing reactions to proceed for 5 min, enzymatic activity was read [3].

#### 2.4.4. Biogenic Amine Production

Biogenic amine (BA) production by LAB isolates was assayed using the Bover-Cid and Holzapfel method [20]. LAB isolates cultivated overnight in MRS (1 mL) were added to 9 mL of MRS containing 0.1% precursor amino acid, histidine, tyrosine, or ornithine, and cultivated for 24 h at 25 °C. Thereafter, 10 μL samples of culture broth were inoculated onto decarboxylase medium plates designed by Bover-Cid and Holzapfel (0.5% tryptone, 0.5% yeast extract, 0.5% meat extract, 0.25% sodium chloride, 0.2% ammonium citrate, 0.2% K_2_PO_4_, 0.1% tween 80, 0.05% glucose, 0.02% MgSO_4_, 0.01% CaCO_3_, 0.005% MnSO_4_, 0.005% pyridoxal-5-phosphate, 0.004% FeSO_4_, 0.001% thiamine, 0.006% bromocresol purple, 2.0% agar; pH 5.3) and containing 1.0% (*v/v*) of each precursor amino acid. Plates were incubated for 48 h at 25 °C, and BA-producing strains were identified by the development of a purple color around colonies.

*Lactobacillus* sp. ATCC 33222™ and *Enterococcus faecalis* ATCC 29212™ were used as positive controls for the production of BA, histamine, and putrescine, and tyramine, respectively [21,22].

#### 2.4.5. Stress Tolerance: Temperature, pH, and NaCl concentration

To determine the effect of temperature on cell viability, the selected LAB isolate was cultivated in MRS broth for 24 h at 25 °C, harvested, resuspended in MRS broth of the same volume (final cell concentration; 9.40 log CFU/mL), and then incubated for 72 h at −2, 0, 4, 10, 20, 30, 50, or 70 °C.

Cell viability of LAB isolate under acid and alkali conditions was also investigated. LAB isolate was cultivated in MRS broth for 24 h at 25 °C, harvested, resuspended in MRS broth adjusted to pH 2.0~10.0 of the same volume, and incubated for 72 h at 25 °C.

To determine the salt tolerance of LAB isolate, LAB isolate cultivated for 24 h was harvested, resuspended in MRS broth containing 1~15% NaCl of the same volume, and incubated for 72 h at 25 °C.

During the 72 h incubation periods of the temperature, pH, and NaCl concentration tolerance experiments, viable cell counts (CFU/mL) were determined every 24 h using a plate counting method [3].

#### 2.4.6. Exopolysaccharide (EPS) Production and Determination of EPS Monosaccharide Composition

LAB isolates cultivated overnight in MRS broth were spotted (3 μL) onto sucrose medium (5.0% sucrose, 1.0% tryptone, 0.5% yeast extract, 0.5% dipotassium phosphate, 0.5% diammonium citrate; pH 7.0), incubated for 48 h at 25 °C, and then EPS production around colonies was observed, as previously described [23].

To purify LAB-produced EPS, the selected LAB isolate was cultured for 48 h at 25 °C in sucrose broth medium and the EPS produced was purified by ethanol precipitation [23]. Briefly, culture was centrifuged (9950× *g*, 25 min, 4 °C), trichloroacetic acid (4%; *v/v*) was added, and then denatured proteins were removed by centrifugation. Supernatant was passed through a 0.4 μm filter (Advantec, Tokyo, Japan) and mixed with two volumes of 95% ethanol for 16 h at 4 °C. EPS was harvested, resuspended in distilled water, and dialyzed (MW cut-off 10,000 Da; Spectrum, Rancho Dominguez, CA, USA) for 24 h at 4 °C. The dialyzed solution obtained was freeze-dried and used for further analysis.

To identify the monosaccharide composition of EPS, purified EPS was hydrolyzed (5 N H_2_SO_4_, 6 h, 100 °C) and analyzed by HPLC (Ultimate 3000, Thermo Scientific Dionex, Sunnyvale, CA, USA) using an Aminex 87H column (300 × 10 mm; BioRad) and an RI detector (Refracto MAX520, Tokyo, Japan). H_2_SO_4_ (0.01 N) was used as the elution buffer at a flow rate of 0.5 mL/min. Gel permeation chromatography (GPC) was performed using a serial set of three columns (Ultrahydrogel 120, 500, and 1000; Waters, Milford, MA, USA). Sodium azide in water (0.1 M) was used as an elution buffer at a flow rate of 1 mL/min. The data obtained were used to determine the molecular weight of the purified EPS using a software package (Chromeleon ver. 6.8, Dionex, CA, USA).

#### 2.4.7. Antimicrobial Activity

Antimicrobial activities of the selected LAB isolate against pathogenic bacteria and food spoilage molds were assayed using the paper disc assay [24]. The microbial strains used and their culture media and culture conditions are listed in Supplementary Table S1. ATCC strains were purchased from the American Type Culture Collection (Manassas, VA, USA), KCCM strains from the Korean Culture Center of Microorganisms (Seoul, Korea), and PF strains were isolated in our laboratory [25].

To prepare culture filtrate of the LAB isolate for antimicrobial studies, LAB was cultivated in MRS broth for 48 h at 25 °C, harvested, and filtered (0.4 μm pore size, Advantec). The resulting filtrate was used as an antimicrobial sample. Simultaneously, the culture filtrate treated with protease (2 mg/mL; Sigma) for 6 h at 37 °C was also used as an antimicrobial sample. Moreover, organic acids produced by LAB isolate was determined by HPLC using the method used for identifying monosaccharides in EPS (Section 2.4.6), and then the organic acid mixture in the culture filtrate quantified was also used as an antimicrobial sample.

Antimicrobial assay and evaluation of antibacterial and antifungal activities were performed using 1X and 5X concentrated antimicrobial samples, respectively. For antibacterial assays, plates were prepared by spreading each pathogen at 6 log CFU/mL on an appropriate medium, and then paper discs (diameter 8 mm; Advantec) were placed on the medium and 100 μL aliquots of antibacterial samples were spotted onto paper discs. For antifungal assays, plates were prepared by adding mold (6 log spores/20 mL of MEA or PDA) to 1.5% (*w/v*) Bacto agar (Duchefa, Harlem, The Netherlands) as listed in Supplementary Table S1. Spore solutions were prepared as previously described [24]. Paper discs (diameter 8 mm; Advantec) on MEA or PDA plates were spotted (100 μL) with the prepared antifungal samples. Plates were incubated for 24~48 h at 25~37 °C, and the diameters of inhibition zones around colonies were measured using a caliper (CD-15CPX, Mitutoyo, Kawasaki, Japan).

### 2.5. Genome Sequencing and Analysis

Genomic DNA extraction was performed using DNeasy Blood & Tissue Kits (Qiagen), and whole-genome sequencing of the selected LAB isolate was performed as previously described [7]. The complete genome of LAB isolate was sequenced by Illumina HiseqXten sequencing and PacBio RS single-molecule real-time sequencing and constructed de novo using the hierarchical genome assembly process; paired-end reads were obtained by Illumina sequencing by Macrogen (Seoul, Korea). The genome sequences of the chromosome and two plasmids have been deposited in GenBank (accession numbers: CP071950~2).

Whole-genome sequences were annotated via the RAST server using the RASTtk scheme (https://rast.nmpdr.org/ (accessed on 10 January 2021)) and the KEGG database using BlastKOALA web tool (http://www.kegg.jp/blastkoala/ (accessed on 12 May 2021)). The general and functional genome features of the LAB isolate were analyzed using RAST annotation and BlastKOALA results. Putative bacteriocin genes were identified using BAGEL 4. Antibiotic resistance genes were analyzed using ResFinder ver. 4.1.

### 2.6. Statistical Analysis

Results were statistically analyzed using the Statistical Package for the Social Science (SPSS) program (Version 26.0 for Window, Chicago, IL, USA) and data were expressed as means ± standard deviations (SD). All experiments were conducted three times with duplicate determinations. Means differences among data were assessed by Duncan’s multiple range test (*p <* 0.05).

## 3. Results and Discussion

### 3.1. Microorganisms Present in Kimchi Samples, and Isolation and Identification of Psychrotropic LAB

**Figure 1:**
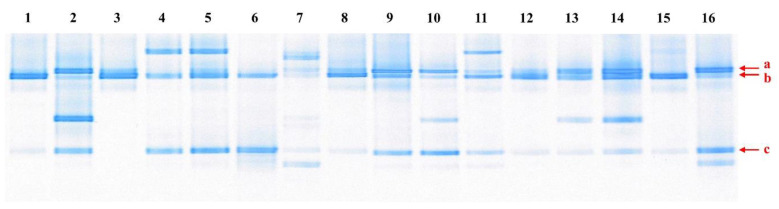
PCR-DGGE patterns of 16S rRNA gene fragments from kimchi samples. 1~16: Kimchi samples 1~16. The closest relatives of the fragments (arrowed) were determined and compared with sequences in GenBank.

Microbial analysis was performed on kimchi samples stored at 0~10 °C for 1~4 months by PCR-DGGE (Figure 1). The microbial profiles of the 16 kimchi samples were slightly different; however, three bands (a~c; arrowed) were detected as main bands in most of the samples. Bands a, b, and c were identified as *Lb. sakei* (with 99.8% identity), *W. koreensis* (with 100% identity), and *Leuconostoc gelidum* or *Leuconostoc inhae* (with 100% identities), respectively. The same results were obtained for kimchi fermented at −1.5~0 °C for 2~3 months in our previous study [7]. Kim et al. also reported that *L. gelidum* is a dominant *Leuconostoc* species in kimchi fermented at 8 °C [26].

Psychrotrophic LAB *Lb. sakei* and *W. koreensis* are frequently detected and isolated from kimchi using a routine isolation method for mesophiles because both can grow well at 0 or 30 °C [10,11]. However, *L. gelidum* or *L. inhae* can barely grow at 30 °C [14,27]. Although *L. gelidum* or *L. inhae* are frequently detected in kimchi using culture-independent methods [6,8], few studies have attempted to characterize these LAB species because they are rarely isolated from kimchi due to the use of 30 °C for isolation experiments [27,28,29].

Kimchi samples, stored at 0~10 °C for 1~3 months, were collected from different locations in South Korea. LAB isolates^1^ were identified by determining 16S rRNA gene sequences (1350~1522 bp) and then compared with those in the NCBI Genbank database^2^. In the present study, we isolated and characterized LAB strains corresponding to band c in Figure 1, which possibly were psychrotrophic strains of *L. gelidum* or *L. inhae*. As shown in Table 1, 23 LAB strains were isolated from the 16 collected samples and identified based on their 16S rRNA gene sequences—that is, 12 strains of *L. gelidum* subsp. *gasicomitatum*, six strains of *L. gelidum* subsp. *aenigmaticum*, two strains of each of *L. inhae* and *Lactobacillus algidus*, and one strain of *Leuconostoc carnosum*, which are all psychrotrophic LAB [30,31]. We selected six strains (LAB 11, 14, 18, 19, 21, and 22) of *L. gelidum* subsp. *aenigmaticum* from the 23 isolates for further investigation, because limited information has been published on *L. gelidum* subsp. *aenigmaticum*.

The selected six LAB isolates were catalase-negative, Gram-positive, and oval cocci-shaped cells and formed glossy, ivory-colored colonies. All six LAB isolates assimilated L-arabinose, ribose, D-xylose, D-glucose, D-fructose, D-mannose, α-methyl-D-glucoside, N-acetyl glucosamine, esculine, cellobiose, maltose, sucrose, trehalose, raffinose, β-gentiobiose, D-turanose, 2-keto-gluconate, and 5-keto-gluconate. On the other hand, the six strains differed in terms of carbohydrate assimilation, e.g., LAB 11 was negative for melibiose utilization and LAB 11 and 14 were negative for gluconate utilization (Supplementary Table S2).

According to a recently published reclassification study, *L. gelidum* comprises three phylogenetically distinct subspecies, *L. gelidum* subsp. *gelidum*, *L. gelidum* subsp. *gasicomitatum*, and *L. gelidum* subsp. *aenigmaticum* [14]. These three subspecies exhibited distinctive phenotypic characteristics with respect to growth in the presence of 6.5% NaCl, heme-stimulated aerobic growth, and acid production from amygdalin, arbutin, and salicin. For example, *L. gelidum* subsp. *aenigmaticum* did not assimilate amygdalin, arbutin, or salicin, but it assimilated ribose. Furthermore, most of the *L. gelidum* subsp. *aenigmaticum* grew in the presence of 6.5% NaCl and the aerobic growth of *L. gelidum* subsp. *aenigmaticum* was not stimulated by heme [14]. As shown in Supplementary Table S2 (indicated by shaded region), the six isolated LAB strains showed distinctive characteristics for *L. gelidum* subsp. *aenigmaticum*.

### 3.2. Growth at Low Temperature

**Figure 2:**
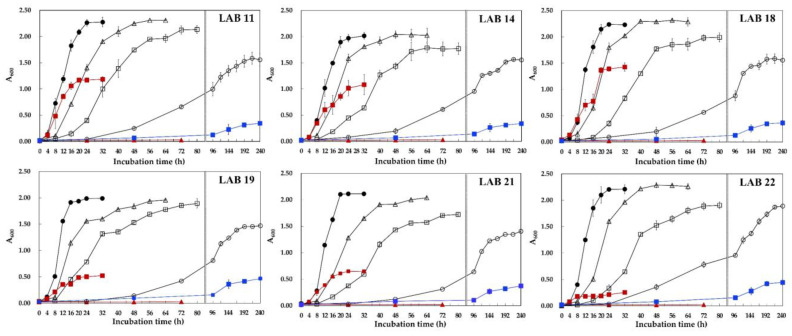
Growth of LAB isolates at different temperatures. LAB isolates were incubated in MRS broth at 0 °C (■), 5 °C (○), 10 °C (□), 15 °C (△), 25 °C (●), 30 °C (■), or 37 °C (▲) for 0~240 h.

Growth of the six selected LAB isolates at 0~37 °C in MRS broth was investigated. Typical of the species, *L. gelidum* grew at 5 °C, but not at 37 °C [14]. As shown in Figure 2, all six LAB grew well at 5 °C and even grew at 0 °C, but not at 37 °C. Maximum cell growth was observed at 15~25 °C, and all six isolates grew better at 5 °C than at 30 °C. Unlike most *Leuconostoc*, *L. gelidum* subsp. *aenigmaticum*, *L. gelidum* subsp. *gasicomitatum*, *L. gelidum* subsp. *gelidum*, *L. inhae*, and *Lb. algidus* are known to have more obligate psychrotrophic characteristics [30].

Furthermore, the growth profiles of the six *L. gelidum* subsp. *aenigmaticum* strains differed with respect to temperature. LAB 22 barely grew at 30 °C (A_600_ ≈ 0.19), whereas LAB 18 grew moderately at 30 °C (A_600_ ≈ 1.43) in MRS broth (Figure 2). When we cultivated the LAB isolates at 30 °C on the MRS plate, no isolate was found to grow (data not shown). Pothakos et al. also reported that most isolates assigned to *L. gelidum* subsp. *gasicomitatum*, *L. gelidum* subsp. *gelidum*, *L. inhae*, *Lactococcus piscium*, and *Lb. algidus* were unable to grow on plates at 30 °C [30], which is widely used in mesophilic enumeration protocols (ISO 4833:2003 and ISO 15214:1998) [32,33]. However, as shown in Figure 2, LAB 19, 21, and 22 showed low growth (A_600_ ≈ 0.19~0.65), and LAB 11, 14, and 18 showed moderate growth (A_600_ ≈ 1.08~1.43) when cultivated in broth at 30 °C.

Psychrophilic and psychrotrophic microorganisms can grow at 0 °C. Psychrophiles have optimal growth temperatures below 15 °C and an upper limit of 20 °C, while psychrotrophs (psychrotolerants) grow optimally at 20~25 °C [15,34]. Our results demonstrate that the six strains of *L. gelidum* subsp. *aenigmaticum* selected showed typical psychrotrophic growth patterns.

### 3.3. Phenotypic Characteristics as Starter Cultures

#### 3.3.1. Sensory Properties

*L. gelidum*, *L. gelidum* subsp. *gelidum*, and *L. gelidum* subsp. *gasicomitatum* are known as spoilage organisms for packaged refrigerated foods, meat, and meat products [14]. These LAB cause food deterioration, package bulging due to CO_2_ formation, buttery off-odors associated with diacetyl and acetoin formation, considerable levels of acidification (e.g., due to acetic acid), green discoloration, and slime formation [35,36]. However, little is known about vegetable spoilage by *L. gelidum* subsp. *gasicomitatum* or *L. gelidum* subsp. *gelidum* [35]. Moreover, *L. gelidum* subsp. *aenigmaticum* has not been reported to be the prevailing community component in any major food spoilage case [14,37].

On the other hand, *Leuconostoc* species including *L. citreum* and *L. mesenteroides* (mesophiles), and *L. gelidum* subsp. *gelidum*, *L. gelidum* subsp. *gasicomitatum*, and *L. inhae* (psychrotrophs) are readily detected in fermented kimchi [2,6]. Furthermore, *Leuconostoc* species, mainly *L. citreum* and *L. mesenteroides*, isolated from kimchi improve the organoleptic quality and are used as starter cultures to improve sensory qualities and extend shelf-life [2]. Meanwhile, psychrotrophic *Leuconostoc* species, which are detectable in kimchi samples using a culture-independent method [8], have never been used as starter cultures for kimchi fermentation due to their infrequent isolations.

In this study, to investigate the sensory attributes of LAB isolates in fermented food, we performed sensory evaluations of kimchi juices fermented using the LAB isolates. Kimchi juices were prepared and fermented using the six selected LAB isolates as starter cultures. The initial pH value of freshly prepared kimchi juice was pH 5.7. After fermentation, the pH values of LAB juices ranged from 4.01 to 4.23, while the pH value of the control juice was 5.5. Sensory evaluations of fermented kimchi juices (Table 2) showed that the sourness of LAB juices increased according to pH values. Furthermore, the sensory qualities of the juices depended on the starters used. In particular, LAB 21 and LAB 22 juices had fresh tastes and pleasant fermented flavors combined with moderate sourness and a carbonated taste, whereas the other four LAB juices had poorer sensory qualities due to a strong off-flavor (a sewerage-like smell) as compared with control juice.

LAB 21 and LAB 22 showed good sensory properties for kimchi juice fermentation, which suggests that they have potential as useful LAB starter cultures for vegetable or fruit fermentation at low temperatures. Based on the above results, we finally selected LAB 22, which exhibited more obligate psychrotrophic characteristics than LAB 21 (in Figure 2), and designated it *L. gelidum* subsp. *aenigmaticum* LS4.

#### 3.3.2. Safety Aspects

Psychrotrophic LAB species have attracted much attention recently [35,36]; however, information about psychrotrophic LAB is lacking. LAB are generally recognized as safe and have a long history of use as starter cultures for various fermented foods [9]. New species and more specific bacterial strains are being sought as novel starter cultures or probiotic candidates; however, the efficacy and safety of new strains must be evaluated.

*L. aenigmaticum* LS4 did not exhibit α- or β-hemolytic activities (data not shown) or any harmful enzyme activities, especially β-glucuronidase and α-chymotrypsin activities (Supplementary Table S3), which may have negative effects in the colon [9]. In API ZYM analysis, *L. aenigmaticum* LS4 presented β-galactosidase activity (≥40 nmol). However, *L. aenigmaticum* LS4 (LAB 22) produced a lactose-negative reaction in the API 50 CHL assay (Supplementary Table S2). In vivo, lactose is hydrolyzed to glucose and galactose by β-galactosidase, and thereafter, glucose and galactose are metabolized to produce acids [9]. Thus, we further evaluated the β-galactosidase activity of *L. aenigmaticum* LS4 and found that its activity was quite low (15.33 Miller units) as compared with other LAB strains (more than 24 Miller units) that produced a lactose-positive reaction in the API 50 CHL assay (data not shown). This result shows that *L. aenigmaticum* LS4 exerts low β-galactosidase activity; in the API 50 CHL assay, its lactose utilization was too low to register as positive (supplementary Table S2).

Antibiotic resistance testing showed that *L. aenigmaticum* LS4 was susceptible to all antibiotics tested, except vancomycin (Table 3). According to the technical guidelines issued by EFSA [19], no breakpoint for vancomycin is required for *Leuconostoc* spp., as the genus *Leuconostoc* is well known to be intrinsically resistant to vancomycin [9].

In the BA production assay, *L. aenigmaticum* LS4 did not produce BA(s), histamine, putrescine, or tyramine from histidine, ornithine, or tyrosine (precursor amino acids). In contrast, *Lactobacillus* sp. ATCC 33222™ and *E. faecalis* ATCC 29212™, which were used as positive controls for BA production, produced BA(s) from precursor amino acids (Supplementary Figure S1). On the other hand, *Leuconostocs* are known to produce BA and tyramine from tyrosine [36].

Based on considerations of the above virulence determinants, *L. aenigmaticum* LS4 does not possess any harmful characteristics such as hemolytic activity, undesirable enzymatic activities, antibiotic resistance, or biogenic amine-producing ability. Thus, it can be reasonably concluded that *L. aenigmaticum* LS4 can be safely used as a starter culture in food or feed.

#### 3.3.3. Technical Aspects

We investigated the heat, salt (NaCl), and acid/alkali tolerances of *L. aenigmaticum* LS4 (Table 4). *L. aenigmaticum* LS4 was stable when exposed to temperatures from −2 to 20 °C (100% survival rate at 48 h). However, no surviving *L. aenigmaticum* LS4 cells were detected after treatment at 50 to 70 °C for 24 h, and its viability was dramatically reduced after exposure to 30 °C for 48 h and no viable cells were detected after 72 h at 30 °C.

*L. aenigmaticum* LS4 was unaffected by 0~5% NaCl for 24 h at 25 °C, but its viability was dramatically reduced at NaCl concentrations > 7%; no viable cells were detected after treatment with 9% NaCl for 72 h. Interestingly, cell viabilities after treatment with 3 to 5% NaCl were significantly greater than after treatment with 0 to 1% NaCl for 72 h. The optimum NaCl concentration range with respect to cell viability after exposure for 72 h was 3 to 5% NaCl (Table 4).

*L. aenigmaticum* LS4 was reasonably stable in the pH range 6.0 to 8.0 for 24 h at 25 °C, but no surviving cells were observed after treatment with pH 2.0 or 10.0 for 24 h (Table 4). Cell viability steadily decreased after 24 h of exposure to pH 4.0, and at 72 h, only 1 to 0.1% of cells remained viable as compared with cells exposed to pH values of 6.0 to 8.0. When we compared the results of other LAB strains, namely the *L. citreum*, *L. mesenteroides*, *Lb. sakei*, and *Lb. plantarum* strains [7,9,38,39], *L. aenigmaticum* LS4 showed similar or slightly weaker acid tolerances than *L. citreum* and *L. mesenteroides* (heterofermentative LAB) but significantly poorer acid tolerances than *Lb. sakei* and *Lb. plantarum* (homofermentative LAB) strains. On the other hand, Jung et al. reported that *Leuconostoc* spp., such as *L. gelidum* subsp. *gelidum* and *L. gelidum* subsp. *gasicomitatum*, are more acid-tolerant than *L. citreum* and *L. mesenteroides*, because *L. mesenteroides* and *L. citreum* are dominant during early fermentation, while *L. gelidum*, *L. gasicomitatum*, and *Lb. sakei* are dominant at the late kimchi fermentation stage, as determined by culture-independent analysis [8]. However, they did not determine the acid tolerances of these LAB. The results of our study indicate that the acid tolerance of *L. aenigmaticum* LS4 is not as high as that of the homofermentative LAB *Lb. plantarum* [7].

We further investigated the effect of low temperature (4 °C) on the cell viability of *L. aenigmaticum* LS4 at low pH (pH 4.0). Cell viability after exposure to 4 °C/pH 4.0 in MRS was significantly greater than that observed at 25 °C/pH 4.0 in MRS and almost the same as (or slightly lower than) that observed at 4 °C/pH 6.5 in MRS. Notably, the cell viability of *L. aenigmaticum* LS4 at pH 4.0 was significantly improved by incubation at 4 °C (3.03 log CFU/mL at pH 4.0/25 °C/72 h vs. 9.10 log CFU/mL at pH 4.0/4 °C for 72 h vs. 9.38 log CFU/mL at pH 6.5/4 °C/72 h) (shaded regions in Table 4). These results show that *L. gelidum* and *L. gasicomitatum* dominate during the late stage of kimchi fermentation [8,13] because kimchi is stored at temperatures below 10 °C for 1~4 months under acidic conditions (pH ~4.0). In other words, this occurred because psychrotrophic *L. gelidum* or L. *gasicomitatum* have lower optimum growth temperatures than the mesophiles *L. citreum* or *L. mesenteroides* [30,40] and better tolerate low temperatures [41]. Stress tolerance results showed that *L. aenigmaticum* LS4 best tolerated temperatures of −2 to 10 °C and that it achieved a 100% survival rate.

LAB encounter various environmental stresses (e.g., thermal, acid/alkali, salt) during food fermentation. LAB intended for use as starter cultures in the food industry must be able to survive and retain their bioabilities under industrial conditions and in fermented food products. The results in Table 4 demonstrate that *L. aenigmaticum* LS4 is a suitable starter culture for vegetable or fruit fermented at temperatures below 10 °C that can maintain thermally instable nutrients or flavors.

#### 3.3.4. Functional Aspects

**Figure 3:**
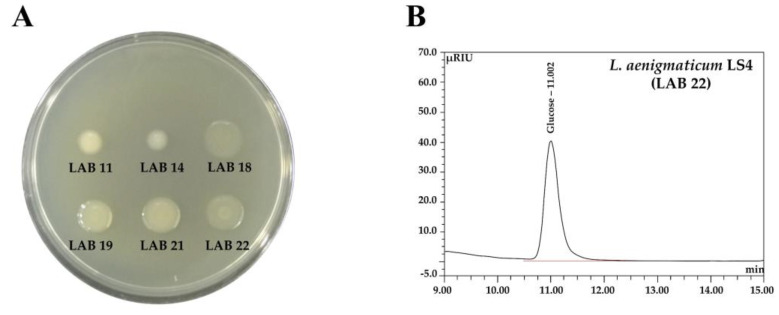
EPS production by LAB isolates. EPS production on a sucrose plate (**A**), and HPLC chromatogram obtained after acid hydrolysis of the EPS produced by *L. aenigmaticum* LS4 (**B**).

EPS production on sucrose medium by the six LAB isolates (LAB 11, 14, 18, 19, 21, and 22) was observed after culture for 48 h at 25 °C (Figure 3) and was greater for LAB 18, 19, 21, and 22 than for LAB 11 and 14. LAB EPSs are important bioproducts that have been shown to improve shelf-life, to enhance techno-functional applications, and to provide health benefits [42]. EPSs can be classified as heteropolysaccharides (HePSs) and monopolysaccharides (HoPSs) based on their monosaccharide compositions and biosynthetic mechanisms [42]. We determined the monosaccharide contents and the molecular weights of the EPS produced by *L. aenigmaticum* LS4 (LAB 22) by HPLC and GPC, respectively. *L. aenigmaticum* LS4 produced EPS composed of glucose only (Figure 3), with molecular weight ≈ 3.7 × 10^6^ Da (data not shown), which is glucan. The molecular weights of the glucans produced by other LAB strains, including *Leuconostoc*, *Lactobacillus*, *Pediococcus*, and *Weissella*, ranged from 10^3^ to 10^7^ Da [43]. EPS is not used as an energy source by producer microorganisms; rather, it protects microbial cells exposed to harsh conditions (e.g., acidic and bilious conditions) [9,40]. We believe that EPS production could improve the techno-functional applications of *L. aenigmaticum* LS4 in the food industry. A considerable number of investigations on the EPSs produced by *Leuconostoc* have been conducted due to their wide-ranging applications in the food, medical, and industrial fields. However, most are related to mesophilic *Leuconostoc* strains such as *L.mesenteroides*, *L. citreum*, or *L. pseudomesenteroides* [44].

As shown in Table 5, L. aenigmaticum LS4 showed strong antibacterial activities against food-borne pathogenic Bacillus cereus, Escherichia coli, Listeria monocytogenes, Pseudomonas aeruginosa, Salmonella enterica serovar. Typhi, and Vibrio parahaemolyticus. L. aenigmaticum LS4 also showed antifungal activities against food spoilage fungi, including Aspergillus flavus, Aspergillus fumigatus, Aspergillus nidulans, Aspergillus ochraceus, and Penicillium roqueforti. L. aenigmaticum LS4 did not exhibit antimicrobial activities against bacteria Micrococcus luteus and Staphylococcus aureus. Its antibacterial activities were stronger than its antifungal activities.

To determine whether its antimicrobial activities were due to the organic acids produced, organic acids produced by *L. aenigmaticum* LS4 in the MRS culture were quantified by IC analysis: 8642.54 mg/L lactic acid, 43.73 mg/L phenyllactic acid, and 14.66 mg/L fumaric acid. Thereafter, the antimicrobial activities of the culture filtrate of *L. aenigmaticum* LS4 and the organic acid mixture in *L. aenigmaticum* LS4 culture quantified were simultaneously assayed. As shown in Table 5, the antibacterial activities of the culture filtrate of *L. aenigmaticum* LS4 were significantly higher than those of the organic acid mixture quantified. However, the antifungal activities of the *L. aenigmaticum* LS4 culture filtrate and the organic acid mixture were almost the same. These results indicated that the antifungal activities of *L. aenigmaticum* LS4 probably originate from the organic acids produced, whereas its antibacterial activities originate from some other antimicrobial(s) (e.g., bacteriocin) and the organic acids produced. We then examined the antimicrobial activities of the culture filtrate treated with protease (Table 5). As was expected, protease treatment did not affect the antifungal activities of the culture filtrate. However, the antibacterial activities of the culture filtrate were significantly reduced by protease treatment and were similar to those of the organic acid mixture. These results confirmed the proteinaceous nature of the bacteriocin produced by *L. aenigmaticum* LS4 and imply its digestion in the gastrointestinal tracts of humans and animals.

EPS production and the antimicrobial activities of *L. aenigmaticum* LS4 as a starter culture would contribute to the health benefits and safety of fermented foods.

### 3.4. General Genome Features and Functional Annotations

The complete genome of *L. aenigmaticum* LS4 consists of a 1,988,425 bp circular chromosome and two circular plasmids (19,308 bp and 11,283 bp), with guanine–cytosine (G + C) contents of 36.8%, 36.7%, and 33.3%, respectively (Table 6, Supplementary Figure S2A). There were 1954 protein-coding genes among a total of 2033 genes and 79 RNA genes on its chromosome, and 23 and 13 coding genes on plasmids 1 and 2, respectively. Twelve and seven single-cutter restriction sites were identified in plasmids 1 and 2, respectively, as determined by NEB cutter v2.0 tool (http://nc2.neb.com/NEBcutter2/ (accessed on 8 January 2021)) analysis. When ccc plasmids 1 and 2 (Supplementary Figure S2B—lane 1) were digested with *Age* I, whose restriction site is located in both plasmids 1 and 2 as a single-cutter site, two linear plasmids of ~19 and 11 kb were produced (Supplementary Figure S2B—lane 2). When plasmids 1 and 2 were digested with *Sph* I or *Nco* I as a single-cutter restriction site predicted on plasmid 1 and 2, respectively, linear plasmid 1 + ccc plasmid 2 (Supplementary Figure S2B—lane 3) and ccc plasmid 1 + linear plasmid 2 (Supplementary Figure S2B—lane 4) were produced. The two circular plasmids of 19,308 bp and 11,283 bp in *L. aenigmaticum* LS4 through the complete genome sequencing were confirmed by these plasmid digestions.

All protein-coding sequences (CDSs) were functionally annotated using the RAST server in 207 subsystems (Supplementary Figure S2C). The results indicated that chromosome genes in *L. aenigmaticum* LS4 were related to protein metabolism (16.08%), carbohydrates (15.69%), amino acids and derivatives (14.14%), nucleosides and nucleotides (10.51%), cofactors, vitamins, prosthetic groups, pigments (8.95%), DNA metabolism (7.13%), fatty acids, lipids, and isoprenoids (5.06%), RNA metabolism (4.54%), and others (Supplementary Figure S2B).

Cold shock protein (CSP), DEAD-box RNA helicase, and ribonuclease (RNase) are commonly known cold-shock response gene families in *L. gasicomitatum*, *Lactococcus piscium*, and *Paucilactobacillus oligofermentans* (formerly *Lactobacillus oligofermentans*), which are all psychrotrophic LAB [45]. In addition, ribosomal protein, tRNA and rRNA modification, and ABC and efflux MFS transporter genes have been suggested to be components of the cold-shock response machinery [45]. In this analysis of genes related to the cold-shock response by psychrotrophic *L. aenigmaticum* LS4, we detected CSP, DEAD-box RNA helicase, and RNase genes as well as genes for efflux ABC transporter, ribosomal protein, and rRNA/rRNA modification (Supplementary Table S4), which have been reported to be related to adaptation or the active growth required for bacterial survival at low temperatures [45,46]. Temperature is one of the major stresses that all living microorganisms must face because food-related bacteria including LAB are repeatedly exposed to low temperatures. The cold-shock-response-related proteins in Supplementary Table S4 have been identified in a variety of microorganisms (not only in psychrotrophs but also in mesophiles and thermophiles) [46], and our analysis using the RAST annotation (https://rast.nmpdr.org/ (accessed on 10 January 2021)) showed that their gene copies were not significantly different. The functionalities of these genes and their machineries should be elucidated by transcriptomic analysis.

For the safety assessment of *L. aenigmaticum* LS4, we investigated genes related to hemolysis, biogenic amine-producing enzyme, and antibiotic resistance. Among the genes related to hemolytic activity, such as putative hemolysin, hemolysin III, and hemolysin A [12], one gene encoding hemolysin III was identified in *L. aenigmaticum* LS4 (Supplementary Table S4), although *L. aenigmaticum* LS4 did not exhibit α- or β-hemolytic activities in this study. KEGG analysis revealed that putative hemolysin-encoding genes are present in many LAB used as probiotics [12]. Antibiotic resistance testing (Table 3) revealed that *L. aenigmaticum* LS4 was susceptible to all antibiotics tested, except vancomycin. One gene for D-alanyl-D-alanine ligase, which is involved in vancomycin resistance, was detected in *L. aenigmaticum* LS4 (Supplementary Table S4). This observation is consistent with a report that *Leuconostoc* species are intrinsically vancomycin-resistant as they harbor D-alanyl-D-alanine ligase, which acts in the synthesis of D-alanyl-D-lactase, which results in a special peptidoglycan structure in *Leuconostoc* species [12]. No genes related to biogenic amine-producing enzymes, including genes for tyrosine-decarboxylase (tyramine-forming), histidine decarboxylase (histamine-forming), ornithine decarboxylase (putrescine-forming), or other amino acid decarboxylases, were detected in *L. aenigmaticum* LS4.

*L. aenigmaticum* LS4 was found to produce HoPS type EPS, composed of glucose only (Figure 3). The synthesis of HoPS is relatively direct when compared with that of HePS, which is typically organized as a cluster with an operon structure [43]. Production of HoPSs is mediated by a glycansucrase encoded by a single gene [42]. In an analysis of the HoPS gene in *L. aenigmaticum* LS4, two different dextransucrases (1,528 and 1,443 amino acids long and with 93.46% and 95.08% identities, respectively) and one levansucrase (1,162 amino acids long with 89.26% identity) were predicted (Supplementary Table S4). When we analyzed the monosaccharide composition of EPS by HPLC (Figure 3), we found that *L. aenigmaticum* LS4 EPS is a glucan comprising a glucose monomer only (Figure 3). Glucan is sub-classified as 1) α-glucan including dextran, alteran, mutan, or reuteran, and 2) β-glucan [42]. This gene analysis indicated that the EPS produced by *L. aenigmaticum* LS4 may be a dextran. The majority of HoPS-producing LAB produce a single glycansucrase enzyme, but some contain more than one glycansucrase and thus may synthesize more than one type of HoPS [42]. These results suggest that one of the two dextransucrase genes predicted to be related to dextransucrase from *L. aenigmaticum* LS4 is functional. The actual gene coding for dextransucrase in *L. aenigmaticum* LS4 should be elucidated by further investigation.

The observed antimicrobial activities of *L. aenigmaticum* LS4 indicate that its antibacterial properties are due to the production of bacteriocin and organic acids (Table 5). BAGEL 4 annotation (Supplementary Table S4) identified two bacteriocin-related genes in *L. aenigmaticum* LS4. When the amino acid sequences of these two putative bacteriocins (71 a.a and 48 a.a) were compared with those of previously identified bacteriocins, one (LS4-1) was predicted to be a penocin A (38.46% identity) and the other (LS4-2) to be an enterocin X chain beta (50.98% identity). LAB bacteriocins can be divided into four classes, though no universally adopted classification scheme exists [47]. Penocin A and enterocin X belong to class IIa bacteriocins (pediocin-like bacteriocins of < 10 kDa) and class IIb (two-peptide bacteriocins), respectively. The majority of class II bacteriocins are biosynthesized as inactive polypeptides harboring a leader peptide with a double-glycine (GG) proteolytic processing site at their N-termini [47]. The polypeptide LS4-1 was 71 a.a long and contained a GG-leader consensus at its N-terminus, and the predicted C-terminal mature part was 50 a.a. When we compared the overall sequences of the polypeptide LS4-1 in *L. aenigmaticum* LS4 with those of the pediocin-like peptides, the polypeptide LS4-1 was found to have slightly different sequences from the consensus sequences of the pediocin-like bacteriocins (red characters in Supplementary Table S5). On the other hand, enterocin X (class IIb) requires two complementary peptides (enterocin X_α_ and X_β_) to exert its antimicrobial activity [47], but the polypeptide LS4-2 was composed of one polypeptide chain, which was 48 a.a. long and contained a GG-leader peptide at its N-terminus, and a predicted mature C-terminal part was 33 a.a. Furthermore, the polypeptide LS4-2 did not contain the GXXXG motifs present in enterocin X_β_ (Supplementary Table S5). These results imply that the bacteriocin from LS4 is a novel bacteriocin belonging to class IIa (pediocin-like). However, further study is required to elucidate its structure.

On the other hand, no functionally annotated results were obtained for the 23 and 13 protein-coding genes on plasmids 1 and 2, respectively, with the exception of one thioredoxin reductase gene in each plasmid.

## 4. Conclusions

Twenty-three psychrotrophic *L. aenigmaticum* strains were isolated as dominant LAB strains along with *Lb. sakei* and *W. koreensis* from kimchi stored at 0 to 10 °C for 1 to 4 months. *L. aenigmaticum* LS4, which showed strict psychrotrophic properties, was finally selected from among the isolates. This strain was found not to present a health risk due to undesirable or virulent properties, and when used in fermented food, it would be expected to improve the sensory qualities and food safety and to provide health advantages due to its production of EPS and antimicrobial compounds (organic acid and bacteriocin). Furthermore, novel insights into the phenotypic and genetic characterization of novel LAB provide insights into new generations of starter cultures. Thus, we conclude that *L. aenigmaticum* LS4 developed in this study possesses the properties required of a functional starter culture for food fermentation at low temperatures. We believe that the present study is the first to characterize *L. gelidum* subsp. *aenigmaticum* based on a complete genome sequence and corresponding phenotypes, and we hope that our results broaden the phenotypic and genomic knowledge regarding the psychrotrophic LAB, *L. gelidum* subsp. *aenigmaticum*.

## Figures and Tables

**Table 1:** Isolation and identification of psychrotrophic LAB from kimchi.

Sampling Location		Kimchi Sample			Isolate				
Province	City/District	Kimchi	Storage Temperature	Storage Period	Strain	Length of 16S rRNA Gene (bp)	Similarity (%)	Identified as LAB ^1^	Accession No. ^2^
Seoul/ Incheon/ Gyeonggi	Seoul	Kimchi 1	0 °C	1 month	LAB 1	1350	100.00	*L. gelidum* subsp. *gasicomitatum*	NR_074997.2
					LAB 2	1517	100.00	*L. gelidum* subsp. *gasicomitatum*	NR_074997.2
	Ganghwa	Kimchi 2	10 °C	3 months	LAB 3	1510	100.00	*L. gelidum* subsp. *gasicomitatum*	NR_074997.2
	Yangpyeong	Kimchi 3	4 °C	2 months	LAB 4	1522	99.46	*Lb. algidus*	NR_028617.1
	Hwaseong	Kimchi 4	4 °C	3 months	LAB 5	1509	100.00	*L. gelidum* subsp. *gasicomitatum*	NR_074997.2
Gangwon	Wonju	Kimchi 5	3 °C ↓	3 months	LAB 6	1467	100.00	*L. gelidum* subsp. *gasicomitatum*	NR_074997.2
Chungcheong	Seocheon	Kimchi 6	0 °C	2~3 months	LAB 7	1468	99.59	*L. gelidum* subsp. *gasicomitatum*	NR_074997.2
					LAB 8	1489	100.00	*L. gelidum* subsp. *gasicomitatum*	NR_074997.2
Daegu/ Busan/ Gyeongsang	Daegu	Kimchi 7	0 °C	2 months	LAB 9	1507	99.93	*L. carnosum*	NR_040811.1
		Kimchi 8	10 °C ↓	3 months	LAB 10	1504	99.66	*L. inhae*	NR_025204.1
	Busan	Kimchi 9	4 °C	3 months	LAB 11	1518	100.00	*L. gelidum* subsp. *aenigmaticum*	NR_133769.1
					LAB 12	1509	100.00	*L. gelidum* subsp. *gasicomitatum*	NR_074997.2
					LAB 13	1512	99.67	*L. gelidum* subsp. *gasicomitatum*	NR_074997.2
	Andong	Kimchi 10	2~3 °C	2~3 months	LAB 14	1501	99.93	*L. gelidum* subsp. *aenigmaticum*	NR_133769.1
					LAB 15	1500	99.66	*L. inhae*	NR_025204.1
	Gimhae	Kimchi 11	5~10 °C	1 month	LAB 16	1519	100.00	*L. gelidum* subsp. *gasicomitatum*	NR_074997.2
					LAB 17	1518	99.33	*Lb. algidus*	NR_028617.1
					LAB 18	1501	100.00	*L. gelidum* subsp. *aenigmaticum*	NR_133769.1
	Uiryeong	Kimchi 12	0 °C	2 months	LAB 19	1479	100.00	*L. gelidum* subsp. *aenigmaticum*	NR_133769.1
Gwangju/ Jeolla	Gwangju	Kimchi 13	6.5 °C	2 months	LAB 20	1519	100.00	*L. gelidum* subsp. *gasicomitatum*	NR_074997.2
	Sunchang	Kimchi 14	4 °C	3 months	LAB 21	1519	99.93	*L. gelidum* subsp. *aenigmaticum*	NR_133769.1
	Iksan	Kimchi 15	0 °C	1 month	LAB 22	1511	100.00	*L. gelidum* subsp. *aenigmaticum*	NR_133769.1
	Jinan	Kimchi 16	0 °C	3~4 months	LAB 23	1429	100.00	*L. gelidum* subsp. *gasicomitatum*	NR_074997.2

Kimchi samples, stored at 0~10 °C for 1~3 months, were collected from different locations in South Korea. ^1^ Identification of LAB isolates by determining 16S rRNA gene sequences (1350~1522 bp). ^2^ Accession No. of 16S rRNA gene sequences of type strain in the NCBI Genbank database.

**Table 2:** Sensory evaluation of kimchi juice fermented using the LAB isolates.

Kimchi Juice	Sourness	Fresh Taste	Carbonate Taste	Pleasant Fermentative Smell	Sewerage-Like Smell	Overall Acceptability
Control juice	1.00 ± 0.00 ^c^	2.00 ± 0.00 ^c^	1.00 ± 0.00 ^b^	1.00 ± 0.00 ^d^	1.22 ± 0.44 ^c^	3.00 ± 0.00 ^c^
LAB 11 juice	4.11 ± 0.14 ^a^	1.89 ± 0.60 ^c^	3.78 ± 0.44 ^a^	2.89 ± 0.33 ^c^	3.44 ± 1.13 ^ab^	2.00 ± 0.87 ^de^
LAB 14 juice	4.22 ± 0.44 ^a^	2.00 ± 0.71 ^c^	3.78 ± 0.44 ^a^	3.00 ± 0.00 ^c^	3.67 ± 0.87 ^ab^	2.00 ± 0.71 ^de^
LAB 18 juice	3.56 ± 0.53 ^b^	1.56 ± 0.73 ^c^	3.78 ± 0.44 ^a^	3.11 ± 0.33 ^c^	4.22 ± 0.67 ^a^	1.44 ± 0.73 ^e^
LAB 19 juice	3.56 ± 0.53 ^b^	2.11 ± 1.05 ^c^	3.78 ± 0.44 ^a^	3.00 ± 0.00 ^c^	3.00 ± 1.32 ^b^	2.22 ± 0.33 ^d^
LAB 21 juice	3.56 ± 0.53 ^b^	4.33 ± 0.50 ^a^	3.78 ± 0.44 ^a^	4.11 ± 0.48 ^a^	1.11 ± 0.22 ^c^	4.78 ± 0.44 ^a^
LAB 22 juice	3.44 ± 0.53 ^b^	3.11 ± 0.33 ^b^	3.78 ± 0.44 ^a^	3.67 ± 0.71 ^b^	1.89 ± 0.93 ^c^	4.00 ± 0.71 **^b^**

Freshly prepared juice was fermented at 25 °C for 24 h without adding starter (control juice) and after adding each LAB isolate (LAB 11~22 juices). Sensory evaluations were carried out using the prepared samples and rated using a 5-point scale for sourness, fresh taste, carbonate taste, pleasant fermentative smell, and sewerage-like smell (1 = very weak, 3 = moderate, and 5 = very strong) and overall acceptance (1 = very bad, 3 = moderate, and 5 = very good). Means with different letters indicate significant differences (*p* < 0.05).

**Table 3:** Minimum inhibitory concentrations (MICs) of antibiotics for *L. aenigmaticum* LS4.

Antibiotics ^1^	AMP	CHL	ERY	GEN	KAN	STR	TET	VAN
Breakpoints for *Leuconostocs* ^2^	2	4	1	16	16	64	8	N.R ^3^
*L. aenigmaticum* LS4	2	4	0.06	0.125	2	4	1	512

^1^ AMP: ampicillin, CHL: chloramphenicol, ERY: erythromycin, GEN: gentamycin, KAN: kanamycin, STR: streptomycin, TET: tetracycline, VAN: vancomycin, ^2^ Breakpoints were as recommended by EFSA (2012) guidelines. ^3^ N.R: Not required.

**Table 4:** Stress tolerance of *L. aenigmaticum* LS4

Stress		Viable Cell Counts (log CFU/mL)			
		0 h	24 h	48 h	72 h
Temperature	−2 °C	9.40 ± 0.04 ^az^	9.44 ± 0.00 ^az^	9.43 ± 0.00 ^az^	9.42 ± 0.01 ^az^
	0 °C	9.40 ± 0.04 ^az^	9.42 ± 0.01 ^az^	9.37 ± 0.03 ^az^	9.37 ± 0.07 ^az^
	4 °C	9.40 ± 0.04 ^azA^	9.46 ± 0.01 ^azA^	9.38 ± 0.04 ^azA^	9.38 ± 0.05 ^azA^
	10 °C	9.40 ± 0.04 ^az^	9.46 ± 0.03 ^az^	9.41 ± 0.00 ^az^	9.36 ± 0.06 ^az^
	20 °C	9.40 ± 0.04 ^az^	9.49 ± 0.06 ^az^	9.47 ± 0.02 ^az^	9.18 ± 0.03 ^by^
	25 °C (Control)	9.40 ± 0.04 ^az^	9.33 ± 0.10 ^az^	8.77 ± 0.04 ^by^	5.06 ± 0.10 ^cx^
	30 °C	9.40 ± 0.04 ^az^	8.82 ± 0.31 ^bz^	4.36 ± 0.48 ^cy^	N.D ^1^
	50 °C	9.40 ± 0.04 ^az^	N.D	N.D	N.D
	70 °C	9.40 ± 0.04 ^az^	N.D	N.D	N.D
pH	pH 2.0	9.40 ± 0.04 ^az^	N.D	N.D	N.D
	pH 4.0	9.40 ± 0.04 ^azA^	8.77 ± 0.07 ^byC^	6.11 ± 0.05 ^cxC^	3.03 ± 0.10 ^cwD^
	pH 6.0	9.40 ± 0.04 ^az^	9.34 ± 0.02 ^az^	8.46 ± 0.12 ^by^	4.94 ± 0.05 ^bx^
	pH 6.5 (Control)	9.40 ± 0.04 ^az^	9.33 ± 0.10 ^az^	8.77 ± 0.04 ^ay^	5.06 ± 0.10 ^bx^
	pH 8.0	9.40 ± 0.04 ^az^	9.37 ± 0.09 ^az^	8.41 ± 0.01 ^by^	6.19 ± 0.27 ^ax^
	pH 10.0	9.40 ± 0.04 ^az^	N.D	N.D	N.D
NaCl	0 % (Control)	9.40 ± 0.04 ^az^	9.33 ± 0.10 ^az^	8.77 ± 0.04 ^ay^	5.06 ± 0.10 ^cx^
	1.0%	9.40 ± 0.04 ^az^	9.37 ± 0.01 ^az^	8.14 ± 0.10 ^by^	5.85 ± 0.40 ^bx^
	3.0%	9.40 ± 0.04 ^azA^	9.19 ± 0.09 ^ayB^	8.60 ± 0.07 ^abxB^	8.56 ± 0.04 ^bxC^
	5.0%	9.40 ± 0.04 ^az^	9.12 ± 0.09 ^ay^	8.51 ± 0.10 ^abx^	8.39 ± 0.06 ^ax^
	7.0%	9.40 ± 0.04 ^az^	8.60 ± 0.08 ^by^	6.82 ± 0.10 ^cx^	3.82 ± 0.31 ^dw^
	9.0%	9.40 ± 0.04 ^az^	4.42 ± 0.50 ^cy^	2.75 ± 0.27 ^dx^	N.D
	12.0%	9.40 ± 0.04 ^az^	3.92 ± 0.13 ^cy^	2.93 ± 0.11 ^dx^	N.D
	15.0%	9.40 ± 0.04 ^az^	4.20 ± 0.05 ^cy^	2.96 ± 0.46 ^dx^	N.D
4 °C, pH 4.0		9.40 ± 0.04 ^azA^	9.31 ± 0.01 ^azB^	9.32 ± 0.05 ^azA^	9.10 ± 0.06 ^ayB^
4 °C, pH 4.0, NaCl 3%		9.40 ± 0.04 ^azA^	9.31 ± 0.03 ^azB^	9.33 ± 0.05 ^azA^	9.19 ± 0.04 ^ayB^

*L. aenigmaticum* LS4 cultivated for 24 h in MRS was harvested and resuspended in fresh MRS broth of the same volume (9.40 ± 0.04 log CFU/mL). Thereafter, *L. aenigmaticum* LS4 tolerances of pH, temperature, or NaCl were determined as described in Materials and Methods. Means with different letters are significantly different (*p* < 0.05) in the same column (a~c), in the same row (w~z), and in the same column, indicated by shading (A~D). ^1^ N.D: Not detected.

**Table 5:** Antimicrobial activities of *L. aenigmaticum* LS4 against molds and bacteria.

Indicator Species		Diameter of Inhibition Zone (mm)		
		Culture Filtrate of LS4 ^1^	Protease Treated Culture Filtrate ^2^	Mixture of Organic Acids ^3^
Molds	*Aspergillus flavus* ATCC 22546™	12.71 ± 0.23 ^a^	12.44 ± 0.08 ^a^	12.53 ± 0.12 ^a^
	*Aspergillus fumigatus* ATCC 96918™	14.19 ± 0.25 ^a^	13.95 ± 0.15 ^a^	13.81 ± 0.17 ^a^
	*Aspergillus nidulans* PF-3	13.31 ± 0.37 ^a^	13.43 ± 0.06 ^a^	13.18 ± 0.18 ^a^
	*Aspergillus ochraceus* PF-2	11.98 ± 0.50 ^a^	11.98 ± 0.16 ^a^	11.88 ± 0.37 ^a^
	*Penicillium roqueforti* ATCC 10110™	10.13 ± 0.53 ^a^	10.67 ± 0.23 ^a^	10.10 ± 0.60 ^a^
Bacteria	*Bacillus cereus* ATCC 14579™	14.47 ± 0.30 ^a^	12.39 ± 0.13 ^b^	12.77 ± 0.24 ^b^
	*Escherichia coli* O157:H7 ATCC 43895™	13.03 ± 0.07 ^a^	12.29 ± 0.29 ^b^	12.39 ± 0.03 ^b^
	*Listeria monocytogenes* ATCC 19113™	12.28 ± 0.26 ^a^	11.43 ± 0.10 ^b^	11.33 ± 0.25 ^b^
	*Micrococcus luteus* ATCC 4698™	0.00 ± 0.00 ^a^	0.00 ± 0.00 ^a^	0.00 ± 0.00 ^a^
	*Pseudomonas aeruginosa* KCCM 11328	16.10 ± 0.25 ^a^	14.68 ± 0.24 ^b^	14.46 ± 0.19 ^b^
	*Salmonella enterica* serovar. Typhi ATCC 14028™	14.72 ± 0.12 ^a^	12.55 ± 0.10 ^b^	12.43 ± 0.30 ^b^
	*Staphylococcus aureus* KCCM 40881	0.00 ± 0.00 ^a^	0.00 ± 0.00 ^a^	0.00 ± 0.00 ^a^
	*Vibrio parahaemolyticus* KCCM 11965	18.17 ± 0.30 ^a^	15.22 ± 0.21 ^b^	14.98 ± 0.18 ^b^

Culture filtrate ^1^ of *L. aenigmaticum* LS4, the culture filtrate treated with protease (2 mg/mL), ^2^ and the organic acid mixture ^3^ in the culture filtrate quantified by IC analysis were used as antimicrobial samples, as described in Materials and Methods. Means with different letters indicate significant differences (*p* < 0.05) between antimicrobial samples.

**Table 6:** General genomic features of *L. aenigmaticum* LS4.

Attribute	Chromosome	Plasmid 1	Plasmid 2
Genome size (bp)	1,988,425	19,308	11,283
G + C content (%)	36.8	36.7	33.3
Total genes (no.)	2033	23	13
Protein coding genes (no.)	1954	23	13
rRNAs (no.)	12	0	0
tRNAs (no.)	67	0	0
GenBank Accession No.	CP071950	CP071951	CP071952

## Data Availability

The data presented in this study are openly available in FigShare at https://doi.org/10.6084/m9.figshare.15081837.v1.

## Supplementary Materials

The following are available online at https://www.mdpi.com/article/10.3390/foods10081899/s1, Figure S1: Biogenic amine production by *L. aenigmaticum* LS4 determined using the screening plate method. Bover-Cid and Holzapfel medium supplemented with 1% ornithine (A), 1% histidine (B), or 1% tyrosine (C). *Lactobacillus* sp. ATCC 33222™ and *Enterococcus faecalis* ATCC 29212™ were used as positive controls, Figure S2: Genomic features of *L. aenigmaticum* LS4. (A) Circular representation of the chromosome and the two plasmid genomes of *L. aenigmaticum* LS4. From the outside circle to the center: CDS on forward strand, CDS on reverse strand, tRNA, rRNA, GC content and GC skew. (B) Agarose gel electrophoresis of plasmids 1 and 2. M, size marker; 1, ccc plasmids 1 and 2; 2, linear plasmids 1 and 2 digested with *Age* I, 3; linear plasmid 1 digested with *Sph* I, 4; linear plasmid 2 digested with *Nco* I (→: ccc plasmid 1, →: ccc plasmid 2, ⇢: linear plasmid 1, ⇢: linear plasmid 2). (C) Subsystem feature of the genomic sequence of *L. aenigmaticum* LS4 analyzed using the RAST server. For the 1983 coding sequences predicted by the RAST server, the subsystem coverage was 30% (the green bar), which contributed to a total of 207 subsystems. The blue bar refers to the percentage of proteins not included in the subsystems, Table S1: Microbial strains subjected to antimicrobial assays with culture conditions, Table S2: Physiological and biochemical properties of LAB isolates, Table S3: Enzymatic activities of *L. aenigmaticum* LS4 as determined using the API ZYM kit, Table S4: Functional annotations of the coding sequences in *L. aenigmaticum* LS4 and their deduced proteins, Table S5: Amino acid sequence alignments of pediocin-like bacteriocins (IIa), two-peptide bacteriocin (IIb), and the predicted bacteriocins from *L. aenimaticum* LS4.
