# Supplementary material for: Characterization of the Psychrotrophic Lactic Acid Bacterium Leuconostoc gelidum subsp. aenigmaticum LS4 Isolated from Kimchi Based on Comparative Analyses of Its Genomic and Phenotypic Properties
Source: Foods. 2021 Aug 16;10(8):1899. doi: 10.3390/foods10081899 (PMC8391443; doi:10.3390/foods10081899)
Supplement: Supplementary file 1 [file foods-10-01899-s001.zip › foods-1297968-supplementary.pdf]

**Supplementary Table S1. Microbial strains subjected to antimicrobial assays with culture conditions**

| Strains | Medium | Culture conditions |
|---|---|---|
| LAB isolate | MRS | 25°C, 48 h |
| Molds | | |
| *Aspergillus flavus* ATCC 22546™ | MEA | 30°C, 48 h |
| *Aspergillus fumigatus* ATCC 96918™ | MEA | 30°C, 48 h |
| *Aspergillus nidulans* PF-3 | MEA | 30°C, 48 h |
| *Aspergillus ochraceus* PF-2 | PDA | 30°C, 48 h |
| *Penicillium roqueforti* ATCC 10110™ | PDA | 25°C, 48 h |
| Bacteria | | |
| *Bacillus cereus* ATCC 14579™ | LB | 37°C, 24 h |
| *Escherichia coli* O157:H7 ATCC 43895™ | LB | 37°C, 24 h |
| *Listeria monocytogenes* ATCC 19113™ | LB | 37°C, 24 h |
| *Micrococcus luteus* ATCC 4698™ | TSB | 30°C, 24 h |
| *Pseudomonas aeruginosa* KCCM 11328 | LB | 37°C, 24 h |
| *Salmonella enterica* serovar. Typhi ATCC 14028™ | LB | 37°C, 24 h |
| *Staphylococcus aureus* KCCM 40881 | TSB | 37°C, 24 h |
| *Vibrio parahaemolyticus* KCCM 11965 | NB+2% NaCl | 37°C, 24 h |

MRS: de Man, Rogosa, and Sharpe; MEA: malt extract agar; PDA: potato dextrose agar; LB: Luria-Bertani; TSB: tryptic soy broth; NB+2% NaCl: nutrient broth containing 2% NB

**Supplementary Table S2. Physiological and biochemical properties of LAB isolates**

| Characteristic | LAB 11 | LAB 14 | LAB 18 | LAB 19 | LAB 21 | LAB 22 |
|---|---|---|---|---|---|---|
| Growth in 6.5% NaCl | + | + | + | + | + | + |
| Heme-stimulated aerobic growth | - | - | - | - | - | - |
| Acid production from: | | | | | | |
| Glycerol | - | - | - | - | - | - |
| Erythritol | - | - | - | - | - | - |
| D-Arabinose | - | - | - | - | - | - |
| L-Arabinose | + | + | + | + | + | + |
| Ribose | + | + | + | + | + | + |
| D-Xylose | + | + | + | + | + | + |
| L-Xylose | - | - | - | - | - | - |
| Adonitol | - | - | - | - | - | - |
| Methyl-BD-xylopyranoside | - | - | - | - | - | - |
| D-Galactose | - | - | - | - | - | - |
| D-Glucose | + | + | + | + | + | + |
| D-Fructose | + | + | + | + | + | + |
| D-Mannose | + | + | + | + | + | + |
| L-Sorbose | - | - | - | - | - | - |
| Rhamnose | - | - | - | - | - | - |
| Dulcitol | - | - | - | - | - | - |
| Inositol | - | - | - | - | - | - |
| Mannitol | - | - | - | - | - | - |
| Sorbitol | - | - | - | - | - | - |
| α-Methyl-D-mannoside | - | - | - | - | - | - |
| α-Methyl-D-Glucoside | + | + | + | + | + | + |
| N-Acetyl glucosamine | + | + | + | + | + | + |
| Amygdaline | - | - | - | - | - | - |
| Arbutin | - | - | - | - | - | - |
| Esculine | + | + | + | + | + | + |
| Salicin | w | - | - | - | - | - |
| Cellobiose | + | + | + | + | + | + |
| Maltose | + | + | + | + | + | + |
| Lactose | - | - | - | - | - | - |
| Melibiose | - | + | + | + | + | + |
| Sucrose | + | + | + | + | + | + |
| Trehalose | + | + | + | + | + | + |
| Inuline | - | - | - | - | - | - |
| Melezitose | - | - | - | - | - | - |
| Rafiinose | + | + | + | + | + | + |
| Starch | - | - | - | - | - | - |
| Glycogen | - | - | - | - | - | - |
| Xylitol | - | - | - | - | - | - |
| β-Gentiobiose | + | + | + | + | + | + |
| D-Turanose | + | + | + | + | + | + |
| D-Lyxose | - | - | - | - | - | - |
| D-Tagatose | - | - | - | - | - | - |
| D-Fucose | - | - | - | - | - | - |
| L-Fucose | - | - | - | - | - | - |
| D-Arabitol | - | - | - | - | - | - |
| L-Arabitol | - | - | - | - | - | - |
| Gluconate | - | - | + | + | + | + |
| 2-Keto-gluconate | + | + | + | + | + | + |
| 5-Keto-gluconate | + | + | + | + | + | + |

The API 50 CHL system was used for carbohydrate assimilation by LAB; w, weakly positive; +, positive; -, negative.

**Supplementary Table S3. Enzymatic activities of *L. aenigmaticum* LS4 as determined using the API ZYM kit**

Unit: nmol

| Enzyme | Activity of *L. aenigmaticum* LS4 |
|---|---|
| Alkaline phosphate | 0 |
| Esterase (C4) | 0 |
| Esterase lipase (C8) | 0 |
| Lipase (C14) | 0 |
| Leucine arylamidase | 10 |
| Valine arylamidase | 0 |
| Cystine arylamidase | 0 |
| Trypsin | 0 |
| α-Chymotrypsin | 0 |
| Acid phosphatase | 0 |
| Naphthol-AS-BI-phosphohydrolase | 5 |
| α-Galactosidase | 10 |
| β-Galactosidase | ≥40 |
| β-Glucuronidase | 0 |
| α-Glucosidase | 20 |
| β-Glucosidase | 0 |
| N-Acetyl-β-glucosaminidase | 0 |
| α-Mannosidase | 0 |
| α-Fucosidase | 0 |

Enzymatic activities indicate nanomoles of hydrolyzed substrate after 4 h of incubation at 25 °C; 0 = no activity, 5 = 5 nmol, and 10 = 10 nmol.

According to the manufacturer's instructions, ≥ or < 20 nmol of substrate hydrolyzed were defined as positive and negative reactions, respectively.

**Supplementary Table S4. Functional annotations of the coding sequences in *L. aenigmaticum* LS4 and their deduced proteins**

| Description | | Strain LS4 | Annotation results | | | |
|---|---|---|---|---|---|---|
| | | CDS length for LS4 (a.a) | CDS length (a.a) | Query coverage (%) | Identity (%) | Predicted protein* |
| Cold-shock response related | Cold shock protein (CSP) | 66 | 66 | 100.00 | 100.00 | *cspA*; cold shock protein |
| | | 74 | 74 | 100.00 | 95.95 | *cspA*; cold shock protein |
| | DEAD-box RNA helicase | 542 | 538 | 100.00 | 98.34 | *deaD*, *cshA*; ATP-dependent RNA helicase DeaD [EC:3.6.4.13] |
| | | 454 | 454 | 100.00 | 99.56 | *cshB*; ATP-dependent RNA helicase CshB [EC:3.6.4.13] |
| | Ribonuclease (RNase) | 116 | 116 | 100.00 | 100.00 | *rnpA*; ribonuclease P protein component [EC:3.1.26.5] |
| | | 518 | 518 | 100.00 | 98.46 | *rny*; ribonucrease Y [EC:3.1.-.-] |
| | | 774 | 774 | 100.00 | 98.32 | *rnr*, *vacB*; ribonuclease R [EC:3.1.13.1] |
| | | 328 | 328 | 94.00 | 91.26 | *rnhC*; ribonuclease HIII [EC:3.1.26.4] |
| | | 600 | 600 | 100.00 | 98.33 | *rnj*; ribonuclease J [EC:3.1.-.-] |
| | | 148 | 148 | 100.00 | 100.00 | *rnhA*, RNASEH1; ribonuclease HI [EC:3.1.26.4] |
| | | 254 | 254 | 100.00 | 93.31 | *rnhB*; ribonuclease HII [EC:3.1.26.4] |
| | | 124 | 124 | 100.00 | 94.35 | *rnhA*, RNASEH1; ribonuclease HI [EC:3.1.26.4] |
| | | 314 | 314 | 100.00 | 97.45 | *rnz*; ribonuclease Z [EC:3.1.26.11] |
| | | 233 | 233 | 100.00 | 99.14 | *rnc*, DROSHA, RNT1; ribonuclease III [EC:3.1.26.3] |
| | | 186 | 186 | 100.00 | 94.09 | *rnmV*; ribonuclease M5 [EC:3.1.26.8] |
| | | 560 | 560 | 100.00 | 99.64 | *rnj*; ribonuclease J [EC:3.1.-.-] |
| | ABC and efflux MFS transporter | 301 | 301 | 100.00 | 98.01 | ABC-2.A; ABC-2 type transport system ATP-binding protein |
| | Ribosomal protein | 49 | 49 | 100.00 | 100.00 | RP-L33, MRPL33, rpmG; large subunit ribosomal protein L33 |
| | | 49 | 49 | 100.00 | 100.00 | RP-L33, MRPL33, rpmG; large subunit ribosomal protein L33 |
| | rRNA/tRNA modification | 253 | 253 | 100.00 | 96.44 | tRNA1Val (adenine37-N6)-methyltransferase [EC:2.1.1.223] |
| | | 295 | 295 | 100.00 | 96.61 | *ksgA*; 16S rRNA (adenine1518-N6/adenine1519-N6)-dimethyltransferase [EC:2.1.1.182] |
| | | 266 | 255 | 95.00 | 96.08 | *truA*, PUS1; tRNA pseudouridine38-40 synthase [EC:5.4.99.12] |
| | | 302 | 302 | 100.00 | 95.03 | *truB*, PUS4, TRUB1; tRNA pseudouridine55 synthase [EC:5.4.99.25] |
| Safety related | Hemolysis | 212 | 212 | 100.00 | 99.06 | *hly*III; hemolysin III |
| | Vancomycin | 377 | 377 | 100.00 | 96.82 | *ddl*; D-alanine-D-alanine ligase [EC:6.3.2.4] |
| Functional related | Sucrase | 1528 | 1526 | 100.00 | 93.46 | Dextransucrase [EC:2.4.1.5] |
| | | 1443 | 1442 | 100.00 | 95.08 | Dextransucrase |
| | | 1162 | 1228 | 100.00 | 89.26 | Levansucrase / Inulosucrase |
| | Bacteriocin | 71 | 60 | 91.00 | 38.46 | Penocin A |
| | | 48 | 55 | 93.00 | 50.98 | Enterocin X chain beta |

* Functional annotations of CDSs for cold shock response and safety related genes were performed using KEGG BlastKOALA. CDSs for sucrase and bacteriocin were annotated using KEGG BlastKOALA and BAGEL 4, respectively.

**Supplementary Table S5. Amino acid sequence alignments of pediocin-like bacteriocins (IIa), two-peptide bacteriocin (IIb) and the predicted bacteriocins from *L. aenimaticum* LS4.**

| Class | Bacteriocin | Leader sequence | | Mature sequence | |
|---|---|---|---|---|---|
| | | Homology (%)* | Sequence | Sequence | Homology (%) |
| | | | | Y**Y** **GNG**V X**C** KXX**C**X**V**DWG A | |
| IIa | Pediocin PA | 16.67 | ------MKKIEKLTEKEMANII**GG** | -KY**Y**---**GNG**V--T**C**GKHS**C**S**V**DWGKATTCIINNGAMAWATGGHQGNHKC | 18.18 |
| | Sakacin A | 22.22 | ------MNNVKELSMTELQTIT**GG** | ARS**Y**---**GNG**V--Y**C**NNKK**C**W**V**NRGEATQSIIGGMISGWASGLAGM---- | 21.95 |
| | Leucocin A | 33.33 | ------MNNVKELSMTELQTIT**GG** | -KY**Y**---**GNG**V--H**C**TKSG**C**S**V**NWGEAFSAGVHRLANG--GNGFW----- | 18.92 |
| | Penocin A | 27.78 | ------MNNVKELSMTELQTIT**GG** | -KY**Y**---**GNG**V--H**C**GKKT**C**Y**V**DWGQATASIGKIIVNGWTQHGPWAHR-- | 38.10 |
| | LS4-1 | - | ---MKSLQDFQTMNHTQLAQVN**GG** | KRV**Y**IPN**GNG**AWLDSNTGKGG**V**DWNVAVPALGSIMVNGWAQNGPLAHLHP | - |
| | | | | **GXXXG motifs** | |
| IIb | Enterocin $X_{\beta}$ | 60.00 | MKKYNELSKKELLQIQ**GG** | IAPIIVAGLGYLVKDAWDHSDQIIS**GFKKGWNGG**RRK-- | 33.33 |
| | LS4-2 | - | ---MNKLSEQELSQIS**GG** | IWPLVVAG--YLGYQAFEHSDQIVAGW----NHAGKKHL | - |

* Predicted bacteriocins in *L. aenigmaticum* LS4 were compared with other bacteriocins using CLUSTAL W (https://www.genome.jp/tools-bin/clustalw). Bold red characters indicated conserved sequences.

| A | | B | | C | |
|---|---|---|---|---|---|
| *Lactobacillus* sp. ATCC 33222™ | *L. aenigmaticum* LS4 | *Lactobacillus* sp. ATCC 33222™ | *L. aenigmaticum* LS4 | *Enterococcus faecalis* ATCC 29212™ | *L. aenigmaticum* LS4 |

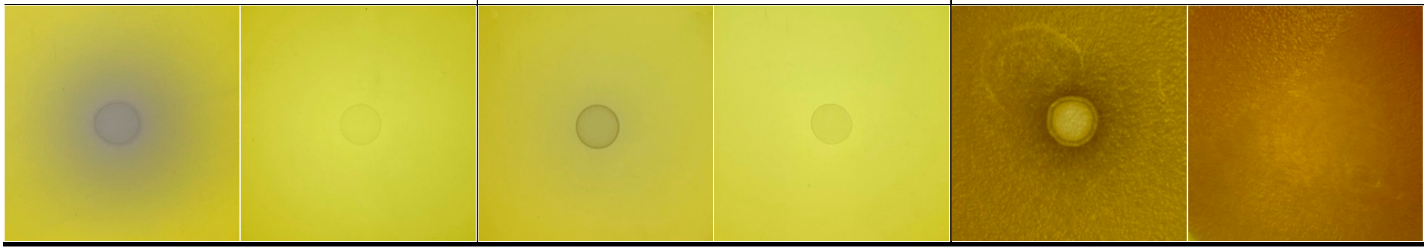

**Supplementary Figure S1. Biogenic amine production by *L. aenigmaticum* LS4 determined using the screening plate method. Bover-Cid and Holzapfel medium supplemented with 1% ornithine (A), 1% histidine (B), or 1% tyrosine (C). *Lactobacillus* sp. ATCC 33222™ and *Enterococcus faecalis* ATCC 29212™ were used as positive controls.**

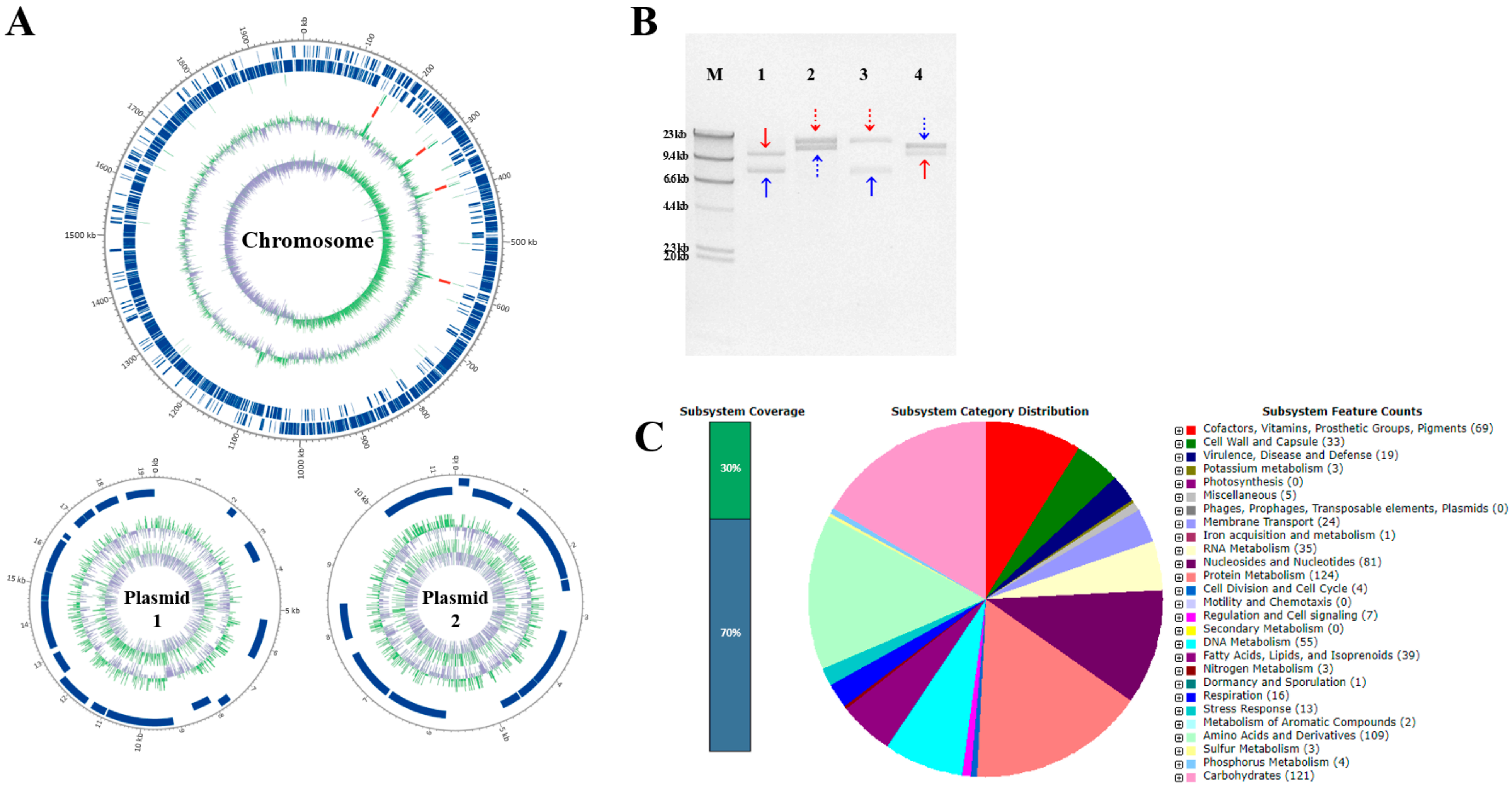


**Supplementary Figure S2. Genomic features of *L. aenigmaticum* LS4. (A) Circular representation of the chromosome and the two plasmids genomes of *L. aenigmaticum* LS4. From the outside circle to the center; CDS on forward strand, CDS on reverse strand, tRNA, rRNA, GC content and GC skew. (B) Agarose gel electrophoresis of plasmids 1 and 2. M, size marker; 1, ccc plasmids 1 and 2; 2, linear plasmids 1 and 2 digested with *Age* Ⅰ, 3; linear plasmid 1 digested with *Sph* Ⅰ, 4; linear plasmid 2 digested with *Nco* Ⅰ (→ : ccc plasmid 1, → : ccc plasmid 2, ⇢ : linear plasmid 1, ⇢ : linear plasmid 2). (C) Subsystem feature of the genomic sequence of *L. aenigmaticum* LS4 analyzed using the RAST server. For the 1983 coding sequences predicted by the RAST server, the subsystem coverage was 30% (the green bar), which contributed to a total of 207 subsystems. The blue bar refers to the percentage of proteins not included in the subsystems.**
